# Home-Based Peanut Oral Immunotherapy for Low-Risk Peanut-Allergic Preschoolers During the COVID-19 Pandemic and Beyond

**DOI:** 10.3389/falgy.2021.725165

**Published:** 2021-09-24

**Authors:** Gilbert T. Chua, Edmond S. Chan, Lianne Soller, Victoria E. Cook, Timothy K. Vander Leek, Raymond Mak

**Affiliations:** ^1^Department of Paediatrics and Adolescent Medicine, The University of Hong Kong, Hong Kong, Hong Kong, SAR China; ^2^Division of Allergy and Immunology, Department of Pediatrics, University of British Columbia, Vancouver, BC, Canada; ^3^British Columbia Children's Hospital Research Institute, Vancouver, BC, Canada; ^4^Community Allergy Clinic, Victoria, BC, Canada; ^5^Pediatric Allergy and Asthma, Department of Pediatrics, University of Alberta, Edmonton, AB, Canada

**Keywords:** peanut allergy, preschooler, OIT, home, Canada

## Abstract

The coronavirus disease 2019 (COVID-19) pandemic has led to the deprioritization of non-emergency services, such as oral food challenges and the initiation of oral immunotherapy (OIT) for food-allergic children. Recent studies have suggested that home-based peanut OIT could be a safe and effective option for low-risk peanut-allergic children. In the period between September 1, 2020, and January 31, 2021, nine preschoolers with a history of mild allergic reactions to peanut underwent home-based peanut OIT. Eight of them (88.9%) completed the build-up phase at home in 11–28 weeks, tolerating a daily maintenance dose of 320 mg peanut protein. During the build-up, six patients (75.0%) reported urticaria, three (33.3%) reported gastrointestinal tract symptoms, and one (14.3%) reported oral pruritis. None of the patients developed anaphylaxis, required epinephrine, or attended emergency services related to OIT. One or two virtual follow-up visits were completed per patient during the build-up phase. Our case series shows that home-based OIT could be offered to the low-risk preschoolers during the COVID-19 pandemic when non-emergency services are limited and could be considered beyond the pandemic, especially for the families living in the rural or remote areas that may otherwise be unable to access OIT.

## Introduction

While oral immunotherapy (OIT) is effective and safe in children allergic to peanut (1–3), the coronavirus disease 2019 (COVID-19) pandemic has led to a delay in non-emergency services, such as oral food challenges (OFCs) and the initiation of OIT (4). The strict avoidance of food allergens or delaying the initiation of food OIT until 12 months of age could lead to increased sensitization and predispose these children to more severe allergic reactions over time, due to progressive intensification of Th2 cytokine expression and an increase in specific immunoglobulin E (sIgE) production in the first few years of life (5, 6). The prolonged elimination of sensitized food in children with atopic dermatitis has been reported to increase the risk of developing acute allergic reactions and anaphylaxis (7–10). Garvey et al. reported a case series of 16 “low-dose—tolerant, high-dose—mild” peanut-allergic children aged 7.0–12.5 years who had received home-based peanut OIT. These children developed mild reactions at doses high enough to exclude them from most OIT protocols. They were advised to start at a peanut protein dose equivalent to one peanut kernel, followed by escalation and doubling the dose every 2 weeks at home till they reached a designated maintenance dose, i.e., the penultimate dose that they reacted in the pre-OIT OFCs. No extra clinic visits were arranged for dose escalation. This strategy was demonstrated to be safe, effective, and welcomed by the families with children having high-dose thresholds during OFC and only mild symptoms (11). Another study by Ball et al. demonstrated that the home-based milk ladders, which some consider a form of OIT, could be safely performed in low-risk young children (12). Preschool-aged children tend to have milder reactions with OIT, with our Canadian real-world study noting the severe reactions occurring in only 0.4% of patients (2). Based on this collective data, we offered home-based peanut OIT to the low-risk preschoolers during the COVID-19 pandemic.

## Methods

The patients from Vancouver, Victoria, and Edmonton, Canada, had peanut allergy as confirmed by the allergist. A baseline OFC was not mandatory due to social distancing measures during the COVID-19 pandemic, unless requested by parents or deemed necessary by the allergist. The preschoolers with documented sensitization to peanut (either by SPT or specific IgE) presenting with grade 1–3 immediate-type hypersensitivity reaction to peanut by history or during an OFC were eligible for home-based OIT in this case series (Table 1) (2). An informed consent was obtained from parents prior to initiation of OIT, as a requirement of routine clinical practice. This case series did not warrant formal ethics review, since the University of British Columbia ethics committee categorized it as quality improvement. Parents were advised to introduce peanut starting from 10 mg peanut protein on a daily basis with a gradual dose increased to 320 mg ~2 weeks apart according to the previously published peanut OIT protocols that showed a low risk of anaphylaxis (2, 13). Patients could start the OIT at a lower dose if the cumulative threshold was ≤10 mg based on OFC. Parents were allowed to use Bamba (Osem Group, Holon, Israel) peanut butter puffs or peanut flour (e.g., powdered peanut butter powder, PB2 Foods, Tifton, GA, USA) and switch to peanut butter at higher doses at their convenience. Prior to starting OIT, the parents received comprehensive education about the recognition and management of adverse reactions, such as anaphylaxis, and an epinephrine autoinjector was always available in case of allergic reactions. The parents could choose to pre-dose non-sedating oral antihistamines before daily doses to prevent mild symptoms and improve dose tolerability (14). Parents were able to extend the interval between dose escalations based on their comfort level. Regular virtual visits every 3 months were provided for follow-up. Parents were advised to record any adverse reactions in a diary that were reviewed during the follow-up. *Ad hoc* virtual communications were arranged in case of non-emergency situations, such as clarification of dose escalation or mild reactions that could be handled by the caregivers at home. The families were advised to use the epinephrine autoinjector and seek immediate medical assessment in the emergency department in case of anaphylaxis.

**Table 1:** Eligible preschoolers for home peanut oral immunotherapy.

**Eligible (need to fulfil all criteria)**
Either failed baseline OFC with cumulative dose <300 mg protein, or objective reactions reported by caregiver with objective evidence of peanut sensitization
Grade 1 to 3 objective allergic reactions to peanut during OFC or reported by caregiver^*^
Objective evidence of peanut sensitization
• SPT ≥ 3mm, and/or
• peanut sIgE ≥ 0.35kU/L
**Ineligible (if fulfil any one criteria)**
Grade 4 severe allergic reaction to peanut during OFC or reported by caregiver^*^
Previous allergic reactions requiring epinephrine to any food
Significant patient or caregiver anxiety preventing initiation of OIT at home
Peanut naïve^#^
Threshold for reaction at baseline OFC less than 10 mg protein
Limited access to emergency care, e.g. patients who live > 30 min from emergency services

* *World Allergy Organization Subcutaneous Immunotherapy Systemic Reaction Grading System (13)*.

# *Suggest introduce peanut at home or arrange observed ingestion/oral food challenge*.

*OFC, oral food challenge; OIT, oral immunotherapy; sIgE, specific immunoglobulin E; SPT, skin prick test*.

## Results

In this study, nine preschoolers aged 9–37 months old who underwent home-based peanut OIT between September 1, 2020, and January 31, 2021, were included. It was found that two (22.2%) children were confirmed peanut-allergic at baseline by open OFC. All the included children experienced objective clinical reactions to peanut (grade 1–2) either during the initial OFC or build-up phase of the home-based OIT. The baseline characteristics and outcomes are listed in Table 2. Eight patients (88.9%) completed the build-up phase in 11–28 weeks, tolerating a daily maintenance dose of 320 mg peanut protein. One patient who started peanut home OIT at 23 months remained in the build-up phase (currently 125 mg) after 24 weeks. The patient developed urticaria while escalating the doses, which was managed with an oral antihistamine as needed. The parents elected to continue with the build-up phase slowly at a pace at which they felt comfortable. During the build-up, six patients (75.0%) reported urticaria, three (33.3%) reported gastrointestinal symptoms, and one (14.3%) reported oral pruritis. None of the patients developed anaphylaxis, required epinephrine, or attended emergency services related to OIT. One or two virtual follow-up visits were completed per patient during the build-up phase.

**Table 2:** Baseline characteristics and Outcomes.

**Age at start of OIT (months)**	**Other food allergies**	**Co-morbidities**	**Pre-OIT peanut first reaction grade, age and symptoms**	**Pre-OIT peanut SPT (mm)**	**Pre-OIT peanut sIgE (kU/L)**	**Cumulative threshold by OFC**	**Progress on last follow up**	**Duration of build-up**	**Immediate reactions during build-up and grade**	**Dose achieved (mg protein)**
13^*^	None	Atopic dermatitis	Grade 2, 5 months—facial urticaria, vomiting	6 mm	N/A	N/A	Completed build-up phase	11 weeks	Grade 1 – Facial urticaria	320 mg
14^**^	Egg	None	Grade 1, 12 months—torso and face urticaria	15 mm	1.3	80 mg	Completed build-up phase	11 weeks	Grade 1 – Facial urticaria	320 mg
37^*^	None	Atopic dermatitis	Grade 1, 6 months old—perioral non-urticarial rash	7 mm	>100	N/A	Completed build-up phase	12 weeks	Grade 1 – occasional pruritis Grade 2 – Abdominal pain	320 mg
9^*^	Milk	Atopic dermatitis	Grade 1, 7 months—Generalized urticaria	12 mm	N/A	N/A	Completed build-up phase	20 weeks	Grade 1 – Facial urticaria	320 mg
9^*^	Egg	Atopic dermatitis	Grade 1, 4 months—Contact Urticaria on the face	7 mm	7.63	N/A	Completed build-up phase	20 weeks	Grade 1 – Abdominal Urticaria	320 mg
12^*^	None	Atopic dermatitis	Grade 1, 6 months—perioral urticaria	8 mm	N/A	N/A	Completed build-up phase	20 weeks	Grade 1 – Facial urticaria	320 mg
12^*^	FPIES to egg	Atopic dermatitis	Grade 2, 7 months—Generalized urticaria, angioedema and vomiting	6 mm	N/A	N/A	Completed build-up phase	28 weeks	Grade 1 – Oral pruritus	320 mg
13^**^	FPIP to dairy	Atopic dermatitis	Grade 1, 11 months—generalized urticaria	4 mm	N/A	150 mg	Completed build-up phase	28 weeks	Grade 1 – Facial urticaria Grade 2 – diarrhea	320 mg
23^*^	None	Atopic dermatitis	Grade 2, 8 months—face, arms and torso urticarial swollen eyelids and ears	4 mm	11.8	N/A	Building-up	In build-up at 24 weeks	Grade 2 – Vomited once; diarrhea during the first 10 doses. Grade 1 – Urticaria with subsequent updosing	125 mg

*FPIES, Food Protein-Induced Enterocolitis Syndrome; FPIP, Food Protein Induced Proctocolitis; N/A, not available; OIT, oral immunotherapy; SPT, skin prick test*.

* *History of objective allergic reactions reported by parents*.

** *Baseline oral food challenges performed*.

*All mg amounts are mg protein*.

## Discussion

The principals of home-based peanut OIT mirror other established practices of home reintroduction of food allergens. An excellent example of home-based reintroduction in infants and preschoolers is the milk ladder for IgE-mediated cow milk allergy, which in a retrospective review of 86 children over a 6-year period showed impressive outcomes with 71 patients (82.5%) having improvement in cow milk tolerance, 26 patients (30.2%) tolerating a normal diet (all dairy products), a high rate of completion (only a further seven patients lost to the program), and no patients experiencing anaphylaxis requiring epinephrine (12). Home-based OIT would be ideally offered to the preschoolers at low-risk of anaphylaxis but not currently offered either OFCs or in-office OIT due to either parent and/or allergist apprehension. Such approaches could minimize the impact on the waitlists of allergists, allowing them to focus on the higher-risk infants and older children for in-person build-ups.

To our knowledge, this is the first case series demonstrating that home-based peanut OIT can be performed safely in low-risk peanut-allergic preschoolers. Although safe and effective food OIT protocols have been published (2, 15), they feature frequent in-person clinic visits for updosing, which are labor-intensive, costly, and disrupt daily family routines because of medical appointments (5). In contrast, this novel approach of using a combination of home OIT updosing with 1–2 virtual assessments for low-risk preschoolers could reduce the healthcare costs and infection risks related to COVID-19 exposure (5). The majority of the patients developed mild grade 1–2 symptoms and none developed anaphylaxis, which is comparable to a recent study on peanut OIT for preschoolers (2). Use of home OIT allowed immediate commencement of therapy, without the need to go on lengthy waiting lists for office visits (e.g., >1 year), thereby minimizing the duration and long-term risks of food avoidance (7, 16).

Home-based peanut OIT could also be considered for home peanut introduction in infants who have been screened for peanut according to the National Institute of Allergy and Infectious Diseases (NIAID) criteria, in situations where the allergist has a very long waiting list for OFCs. The same protocol of gradual escalation used for home-based OIT could potentially be used in other primary or secondary prevention scenarios to minimize any delay in early peanut introduction. One scenario is high-risk infants with severe eczema and/or egg allergy, where there is caregiver hesitancy for either screening SPT/sIgE or in-office OFC. A second scenario is an infant who has already been screened by a primary care provider and has a positive sIgE but is on a lengthy waitlist for allergist SPT. A third scenario is an infant who has had positive screening SPT with an allergist but is on a lengthy waitlist for OFC either with the same allergist or with another allergist who offers infant OFCs (17, 18). The initial NIAID guidelines released in 2017 recommended a screening approach before peanut introduction in infants with severe eczema and/or egg allergy (19). In the real world, however, there are logistical, cost, and resource implications to the clinical practices (20). SPT and sIgE have good negative but poor positive predictive values, and eczematous patients often produce high total and specific IgEs (19, 21, 22), therefore the frequent testing results in the overdiagnosis of food allergies (23). The supervised OFCs are essential for the confirmation of food allergies. However, in practice, OFCs are not consistently accessible. A recent study has shown that fewer allergists would offer OFCs to infants below 12 months old than the older children, (17) and some allergists would only offer SPT but not OFCs for a definitive diagnosis, (18) therefore leading to unnecessary avoidance at least in some infants with positive testing prior to peanut consumption. Lower risk infants may also undergo screening “creep" due to parental anxiety, physician concern, and overdiagnosis of severe eczema, and panel skin testing to multiple foods could lead to unnecessary and prolonged delay in the introduction of peanut and other foods while waiting for OFCs (24). The waiting time for allergist consultation, OFC, and OIT in countries, such as Canada can be multiple years (4, 25), and resources to provide allergy services in the different regions around the world vary (26, 27). These practical issues could lead to unnecessary and prolonged avoidance and increase the risk of developing food allergies, thus compromising the ability to implement early introduction for many children.

More recent guidance has suggested a universal patient-preference-sensitive approach to home peanut introduction during infancy (28). However, home peanut introduction without screening has been associated with superior population health and economic outcomes and may be preferred by many families wary of food allergy overdiagnosis resulting from a surrogate diagnosis made by detecting the peanut sensitization alone but lacking a history of IgE-mediated food reaction (29–31). Incorporating home OIT for selected patients appears to be cost-effective. This case series demonstrated as a proof of concept that the low-risk peanut-allergic preschoolers could build up their peanut doses at home safely and successfully reached maintenance dosing in 11–28 weeks supported by virtual visits without the need for clinic visits in person. In a recent analysis of health and economic outcomes of preschool peanut OIT, use of home OIT dose escalations following OIT clinic initiation was cost-effective unless the risk of a severe OIT reaction at home increased significantly (clinic OIT demonstrated the highest health and economic outcomes if the risk of home OIT anaphylaxis fatality increased beyond 62-fold or anaphylaxis hospitalization costs for home OIT reactions were inflated more than 30%) (32). A recent study has also demonstrated that home peanut introduction combined with virtual physician support was safe and well-received by parents (33, 34).

### Limitations of This Study

These outcomes of our case series should be interpreted with caution due to the small number of patients included in this report. Selection bias is possible, as the included families in this case study were highly motivated to undergo OIT at home, suggesting psychosocial and family factors should be considered. The studies that include larger numbers of patients to further define the preschoolers who would be benefited from home OIT are warranted to confirm the efficacy and safety of this approach.

## Conclusions

There is no consensus on the management of low-risk peanut-allergic children. Some allergists advise strict avoidance, while some may recommend regular ingestion in various quantities (35). The recent literature acknowledges that the majority of the patients with peanut allergy react at higher thresholds and never experience severe reactions (35). As younger children with food allergy tend to have milder reactions than older children (36, 37), our preliminary work shows that home-based OIT could be offered to low-risk peanut-allergic preschoolers earlier during the COVID-19 pandemic when non-emergency services were limited and could be considered beyond the pandemic. Careful patient selection and shared decision-making with families (35) will be needed when offering home OIT to these children. In the areas where all allergists do not feel comfortable offering OIT and access is limited, home-based OIT should be limited to the practices that have ample experience with it and have the resources to address the unforeseen challenges as they come up. In the case of distance, an experienced allergist could partner/collaborate with a local provider to guide OIT. Alternatively, a hybrid office-based initiation of the first few doses followed by home-based escalation could be considered.

## Data Availability Statement

The original contributions presented in the study are included in the article/supplementary material, further inquiries can be directed to the corresponding author/s.

## Ethics Statement

This case series did not warrant formal ethics review, since the University of British Columbia Ethics Committee categorized it as quality improvement.

## Author Contributions

GC drafted the manuscript. EC and LS are responsible for the conceptualization and design of the study. VC, RM, and TV are responsible for the data collection. All authors critically appraised and reviewed the manuscript.

## Funding

This study was supported by the University of Hong Kong Seed Fund for Basic Research for New Staff (Project code: 202009185024). The funding source was not involved in the study design, in the collection, analysis, and interpretation of data; in the writing of the manuscript; and in the decision to submit the manuscript for publication.

## Conflict of Interest

EC has received research support from DBV Technologies; has been a member of advisory boards for Pfizer, Pediapharm, Leo Pharma, Kaleo, DBV, AllerGenis, Sanofi Genzyme, Bausch Health, and Avir Pharma; is a member of the healthcare advisory board for Food Allergy Canada; and was co-lead of the CSACI oral immunotherapy guidelines. VC has been a member of advisory boards for Sanofi Genzyme, Bausch Health, and ALK and has received honoraria from Aralez Pharmaceuticals and CSL Behring. TV has served on advisory boards and received honoraria from Aralez, Bausch Health, and Pfizer. RM is the advisory board member of ALK and Sanofi and has received speaker honoraria from Novartis, Pediapharm, and Astrazeneca. The remaining authors declare that the research was conducted in the absence of any commercial or financial relationships that could be construed as a potential conflict of interest.

## Publisher's Note

All claims expressed in this article are solely those of the authors and do not necessarily represent those of their affiliated organizations, or those of the publisher, the editors and the reviewers. Any product that may be evaluated in this article, or claim that may be made by its manufacturer, is not guaranteed or endorsed by the publisher.

## Abbreviations

OFC: oral food challenge
OIT: oral immunotherapy
sIgE: specific immunoglobulin E
SPT: skin prick test.
